# Supplementary material for: A Novel Probiotic-Based Oral Vaccine against SARS-CoV-2 Omicron Variant B.1.1.529
Source: Int J Mol Sci. 2023 Sep 11;24(18):13931. doi: 10.3390/ijms241813931 (PMC10530581; doi:10.3390/ijms241813931)
Supplement: Supplementary file 1 [file ijms-24-13931-s001.zip › ijms-2580318-supplementary.pdf]

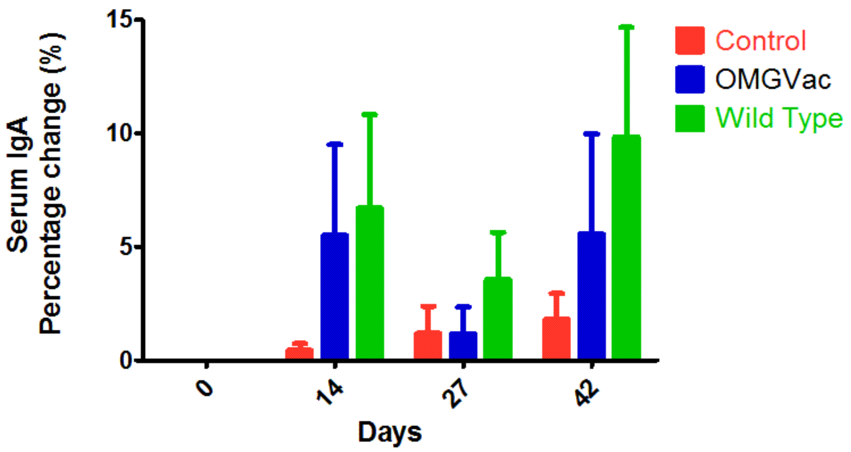

Figure S1. Percentage change in serum IgA level of vaccinated hamsters. No significant difference was observed between groups and days ($p>0.05$).
